# Surgical Complications of Typhoid Fever: First National Typhoid Conference in Niamey, Niger

**DOI:** 10.12688/gatesopenres.16348.1

**Published:** 2025-05-07

**Authors:** Harissou Adamou, Abdoulaye Maman Bachir, Yakoubou Sanoussi, Katherine Shafer, Leah Sukri, Laura Hobbs, Saidou Adama, Amadou Magagi Ibrahim, Ide Kadi, Zabeirou Oudou Abdou Aliou, Abdou Soley Aboul Aziz, Boubacar Moctar, Mahamadou Doutchi Altine, Brah Souleymane, Adehossi Eric, Joseph Emalieu Toko, Mamadou Saidou, Assan Abdoul Nasser, Lassane Kabore, Kathleen Neuzil, Rachid Sani

**Affiliations:** 1 Hôpital National de Zinder, Zinder, Niger; 2 Hôpital National de Niamey, Niamey, Niger; 3 Hôpital de la SIM, Galmi, Niger; 4 University of Maryland Baltimore, Baltimore, Maryland, 21201, USA; 5 Hôpital Général De Reférence, Niamey, Niger; 6 Centre Hospitalier Régional, Tahoua, Niger; 7 Centre Hospitalier Régional, Maradi, Niger; 8 Hôpital National Amirou Boubacar Diallo, Niamey, Niger; 9 University of Yaounde, Soa, Cameroon; 10 Université Abdou Moumouni de Niamey, Niamey, Niger; 11 Ministry of Public Health, Population and Social Affairs, Niamey, Niger; 12 Center for Vaccine Innovation and Access, PATH, Seattle, Washington, 98121, USA

**Keywords:** Typhoid, typhoid intestinal perforation, complication, Africa, Niger, typhoid conjugate vaccine

## Abstract

Typhoid intestinal perforation (TIP) is a life-threatening, late complication of typhoid fever that disproportionately impacts children in low resource settings and continues to have devastating consequences worldwide. Despite elimination of typhoid fever in most high income countries, typhoid fever and TIP remain endemic in many countries around the world as a result of inadequate investments in water, sanitation, and hygiene (WASH) and lack of access to vaccines. A first National Typhoid Conference was held in Niamey, Niger on July 22, 2023, where surgeons and other medical and health professionals from Niger convened with local and international health professionals to discuss their experiences with TIP and advocate for better prevention and treatment of the disease. The high number of intestinal perforations diagnosed during surgery, and the lack of capacity for performing blood cultures motivated surgeons in Niger to convene and share data on complications of typhoid, epidemiology, and diagnosis. TIP, a leading cause of peritonitis in Africa, often results in emergency surgery and has reported mortality rates up to 30% in pediatric patients. The availability of four safe and effective typhoid conjugate vaccines, two with committed financial support from Gavi, the Vaccine Alliance, makes prevention through vaccination a realistic near-option for typhoid fever to complement improvements in WASH.

## Introduction

Typhoid fever is a bacterial infection caused by *Salmonella* enterica serovar Typhi ( *S.* Typhi) that disproportionately impacts the economically disadvantaged. The Global Burden of Disease Study estimates more than seven million cases of typhoid fever and more than 93,000 resulting deaths globally in 2021. ^1^ Typhoid fever and specifically TIP, a late complication of untreated typhoid fever, can be financially catastrophic for families and complications can be difficult to manage.

Patients with TIP often present after about two weeks of initial symptoms, characteristically fever and diffuse abdominal pain. Although clinical signs and symptoms are typically non-specific, the operative finding of at least one oval-shaped perforation on the anti-mesenteric side of the bowel is pathognomonic of TIP and allows surgeons to diagnose typhoid in the operating room. Perforations and pre-perforations occur most commonly in the distal ileum but can occur anywhere in the small intestine or colon. Typhoid may also affect the gallbladder and lead to inflammation and necrosis. TIP remains a major cause of morbidity and mortality in sub-Saharan Africa, with an estimated case fatality rate of 20% overall and up to 30% in children. ^2^

In 2018, the World Health Organization (WHO) recommended programmatic use of typhoid vaccines for the control of typhoid fever. Typhoid conjugate vaccines (TCVs) should be prioritized in countries with the highest burden of disease and/or antimicrobial resistant *S.* Typhi. ^3^ It remains challenging for many countries to introduce this critical vaccine given competing health priorities and the difficulty in estimating the true burden of disease. Diagnosing typhoid fever is complicated due to the non-specificity of symptoms and limited diagnostics in many typhoid endemic countries. Using TIP as a surrogate indicator for typhoid could fill a much-needed disease burden gap that would allow governments to make decisions on TCV introduction for the most marginalized children in rural and remote areas without blood culture capability.

## Justification and meeting goals

Niger, a Francophone country in West Africa, has a population of more than 24 million people. The gross domestic product of Niger is approximately $14.92 billion USD, with an average household income of 338,000 West African CFA franc (approximately $554 USD based on 2024 currency conversion rates) per household. ^4^ Currently, typhoid fever is estimated to impact 333 per 100,000 persons each year in Niger, although many believe this is a large underestimate. ^5^ Surgeons across Niger consistently report high levels of ileal perforations that overwhelm operating rooms and hospitals during the peak typhoid season and are presumed to be typhoid-related. Although there is growing concern within the Nigerien surgical community regarding TIP, access to published data from Niger, like many Francophone African countries, remains limited often due to French publications in local journals, or journals not well-known to non-Nigeriens and Westerners. ^6, 7^

On July 22, 2023, surgeons from across Niger, along with local and international typhoid experts and representatives from the Ministry of Health including the Director of Immunizations, Dr. Assan Abdoul Nasser, convened in Niamey, Niger for the first National Typhoid Conference ( Table 1).

**Table 1.** List of presentations.

Author Order	Presenter Name	Title and Affiliation	Presentation Title (Original French)	Presentation Title (Translated English)
1	Harissou Adamou	MD, Prof Agrégé, Chirurgie Générale, Doyen FSS-UAS, Chef du service de chirurgie B HNZ-Niger (Zinder National Hospital)	Typhoid intestinal perforation: a proposition of a new score prognostic in poor-resource	Typhoid Intestinal Perforation Prognostic Score in Poor-Resource Settings ^12^
2	Abdoulaye Maman Bachir	MD, Chirurgie Générale, Maitre-assistant FSS-UDDM, Chef service urgences chirurgicales HRM-Niger (Hopital National de Niamey)	Indications, complications et poids socio-économiques des stomies digestives au centre Hospitalier Régional de Maradi	Indications, complications and socio-economic weights of digestive stomas at Regional Hospital Center of Maradi ^13^
3	Yakoubou Sanoussi	MD, Chirurgie Générale, Directeur Hôpital Galmi-Niger	Mortalité évitable par la vaccination chez les patients en chirurgie pédiatrique dans les zones rurales du Niger	Vaccine-preventable mortality among pediatric surgery patients in rural Niger
4	Katherine Shafer	MD, FACS, FCS (ESCA), Chirurgie Générale, Chef du service de chirurgie, Hôpital de la SIM Galmi-Niger	Le risque pour les enfants nigériens lié aux perforations intestinales typhiques maternelles (PIT): Une série de cas de complications liées à la grossesse en raison d'une typhoïde sévère	The risk to Nigerian children associated with maternal typhoid intestinal perforations (TIP): A case series of pregnancy-related complications due to severe typhoid
5	Saidou Adama	MD, Chirurgie Générale, Assistante FSS-UAM, Chef service chirurgie viscérale HGR-Niamey (Hôpital Général De Reférence)	Prise en charge de la péritonite aigue à l'Hôpital Général de Référence de Niamey, quelle place occupe la perforation iléale d’origine infectieuse?	Where does ileal perforation of infectious origin fit in the overall management of acute peritonitis at the Niamey General Referral Hospital?
6	Amadou Magagi Ibrahim	MD, Chirurgie Générale, Maitre-assistant FSS-UAS, HNZ-Niger (Zinder National Hospital)	Perforation typhique de l’intestin grêle, un fardeau chirurgical au Niger: cas des régions de Maradi et Zinder	The burden of typhoid perforation of the small intestine in Niger ^14^
7	Ide Kadi	MD, Chirurgie Générale, HNN-Niamey (Hopital National de Niamey)	Les facteurs associés à la morbi-mortalité liée au traitement chirurgical des perforations iléales non traumatiques à l’Hôpital National de Niamey	Factors associated with morbidity and mortality related to the surgical treatment of non-traumatic ileal perforations at the Niamey National Hospital
8	Zabeirou Oudou Abdou Aliou	MD, Chirurgie Viscérale, HGR-Niamey (Hôpital Général De Reférence)	Quelle place pour la cœlioscopie dans prise en charge des perforations iléales d'origine typhique (PIT): présentation de 2 rapports de cas et une revue de la littérature	What role does laparoscopy have in the management of typhoid ileal perforations (TIP): 2 case reports and a review of the literature
9	Abdou Soley Aboul Aziz	MD, Chirurgie Générale, CHR Tahoua-Niger (Centre Hospitalier Régional)	Les péritonites par perforation typhique au CHR de Tahoua	Peritonitis caused by typhoid perforation at the Tahoua Referral Hospital Center
10	Boubacar Moctar	MD, Chirurgie Générale, CHR Maradi-Niger (Centre Hospitalier Régional)	Les péritonites par perforations iléales d’origines typhiques probables au CHR de Maradi: aspects épidémiologiques, thérapeutiques et pronostiques à propos de 858 cas	Peritonitis due to Ileal perforation of probable typhoid origin at the Maradi Referral Hospital Center: epidemiological, therapeutic and prognostic aspects in 858 cases
11	Mahamadou Doutchi Altine	MD, Prof Agrégé, Infectiologie, FSS-UAS, Chef du service maladies infectieuses HNZ-Niger (Zinder National Hospital)	Épidémiologie diagnostic et traitement de la typhoïde Modérateur	Epidemiology, diagnosis and treatment of typhoid fever
12	Brah Souleymane	MD, Prof Agrégé, Médecine interne, FSS-UAM, Directeur général HNABD-Niger (Hôpital National Amirou Boubacar Diallo)	Immunologie et vaccinologie de la fièvre typhoïde	Immunology and vaccinology of typhoid fever
13	Adehossi Eric	MD, Prof Titulaire, Médecine Interne, FSS-UAM, Directeur Général HGR-Niger (Hôpital Général De Reférence)	Vaccin conjugué contre la typhoïde (VCT) Modérateur	Typhoid conjugate vaccine (TCV)
14	Lassane Kabore	PharmD, Ph.D.in Global Health, Senior Program Officer, Center for Vaccine Innovation and Access, Vaccine Implementation Team/Africa	Le vaccine conjugés contre la typhoïde et la consortium TyVAC	The typhoid conjugate vaccine and the TyVAC Consortium
15	Kathleen Neuzil	MD, MPH, FIDSA, Professor of Medicine and Pediatrics, University of Maryland, School of Medicine	Présentation du vaccin conjugué	Conjugate vaccine overview
16	Rachid Sani	MD, Prof Titulaire, Chirurgie Générale, Doyen FSS-UAM, Chef du service de chirurgie A HNN-Niger (Hopital National de Niamey)	Complications chirurgicales de la fièvre typhoïde Modérateur; Commentaires et étapes suivantes sur l’introduction du vaccin au Niger	Surgical Complications of Typhoid Fever. Comments and next steps on vaccine introduction in Niger.
	Mahamadou Doutchi Altine	MD, Prof Agrégé, Infectiologie, FSS-UAS, Chef du service maladies infectieuses HNZ-Niger	Epidémiologie, Diagnostic, Traitement de la fièvre typhoïde	Epidemiology, diagnosis, and treatment of typhoid fever
NA	Mamadou Saidou, MD, Professor	MD, Professor, Recteur de l’Université Abdou Moumouni Dioffo de Niamey	Moderator for Epidemiology, Diagnostic, and Treatment of Typhoid	Moderator for Epidemiology, Diagnostic, and Treatment of Typhoid

With three safe and effective TCVs prequalified and two eligible for introduction support from Gavi, the Vaccine Alliance (Gavi), surgeons came together to share data and experiences to inform country and regional decision-making on TCV introduction. The meeting goals were to share data regarding typhoid burden and complications in Niger, especially those requiring surgical intervention, and to improve care for patients with typhoid and develop strategies to advocate for TCV introduction in Niger.

## Diagnosis and management of typhoid fever in Niger

While blood culture diagnosis is the gold standard for diagnosis of typhoid fever, many low- and middle-income countries face resource limitations and lack the laboratory capacity and capability to diagnose typhoid using this mechanism. Other diagnostics, such as the Widal test, are often inaccurate. As a result, typhoid is often underreported and/or treatment is incorrect, which leads to an increase in severe clinical outcomes. New cost-effective rapid diagnostics are in development but require further testing in endemic settings.

At the Niger meeting, Dr. Mahamadou Doutchi outlined general trends in the diagnosis and treatment of patients with suspected typhoid fever in Niger. Due to the non-specific symptoms of typhoid fever, along with similar seasonal patterns to malaria, many patients are initially treated for malaria, and some will additionally receive antibiotics, oral ciprofloxacin being the most prescribed in the outpatient setting. Many patients worsen and develop abdominal pain, leading to hospitalization and surgery. Only in the operating room, where pathognomonic findings for TIP are found, do many patients receive a definitive diagnosis of typhoid fever. As a result, typhoid fever is commonly referred to as a “surgical disease” in Niger.

Once admitted, patients will generally receive intravenous antibiotics, most commonly a combination of ceftriaxone and metronidazole. A patient’s hospital course can vary due to complications.

## Regional burden of typhoid intestinal perforation: Maradi, Niamey, Tahoua, Zinder

**Figure 1.**
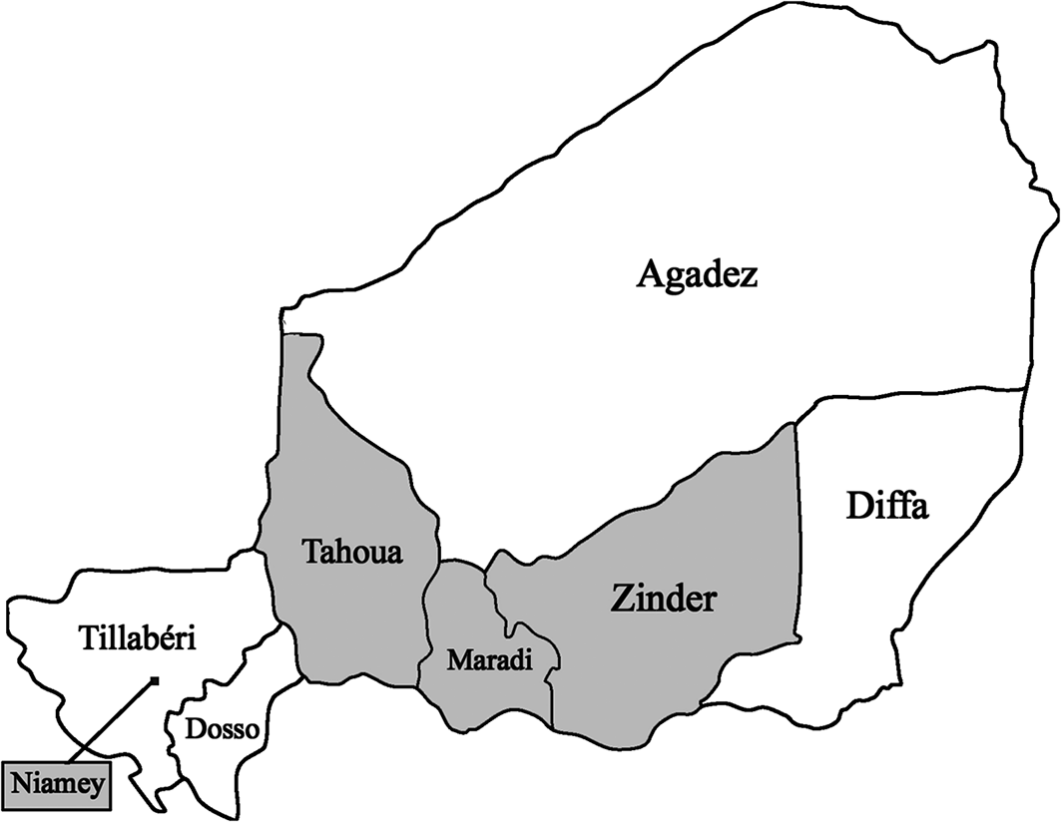
Map of the eight administrative districts of Niger; gray represents the regions with presentations in this summary. This map is a modified version of Niger Regions.png from Wikimedia Commons ( this file), licensed under the Creative Commons Attribution-Share Alike 3.0 Unported license.

There are eight distinct administrative regions in Niger: Agadez, Diffa, Dosso, Maradi, Niamey, Tahoua, Tillabéry, and Zinder ( Figure 1). Information from four of these regions was presented at the conference. Each region was represented by practicing surgeons from their region at the conference, but not all regions came with a presentation.

### Maradi

Presented by Dr. Boubacar Moctar, a retrospective study at Regional Hospital Center (CHR) Maradi was conducted from January 2022 to June 2023. During this time, 858 cases of peritonitis – comprising 38% of all emergent operations at the hospital – were due to ileal perforation. A total of 70% of the cases occurred in patients under the age of 15 years, and 95% originated from rural regions with poor access to healthcare. The most common procedure was small bowel resection with ileostomy creation, occurring in 94% of all operations for ileal perforation. The complication rate was 42% and mortality rate 11.8%.

Dr. Bachir highlighted his recent work titled “Indications, Complications, and Socio-Economic Weights of Digestive Stomas at Regional Hospital Center of Maradi”. ^8^ Between 2019 to 2020, 264 ostomies were created at CHR Maradi, with 84% in TIP patients. A total of 73% of patients who received an ostomy were under the age of 15 years. Although patients received an ileostomy reversal on average four months after their surgery, 71% of children lost at least one year of schooling. Additionally, 51% of patients reported complications associated with their ostomy, the most common being skin irritation around the stoma site. Mortality rates among patients with ostomies were high, at 12%.

### Niamey

Dr. Saidou Adama presented a retrospective study of pediatric acute peritonitis at the general reference hospital of Niamey. Between 2018 and 2023, TIP was the most common intraoperative diagnosis, occurring in 38% of all pediatric peritonitis cases. Primary repair was the most common surgical treatment, while ileostomy was the second most common treatment. Half of all surgical mortality during this time was attributed to TIP. Laparoscopy was presented by Dr. Zabeirou Oudou Abdou Aliou as a reasonable surgical approach for TIP. Two patients with TIP were treated with a laparoscopic approach and experienced no reported complications. The moderator Dr. Rachid Sani did note that one should be cautious with the universal application of laparoscopy in TIP patients.

A prospective study on TIP between 2014 and 2015, presented by Dr. Ide Kadi, demonstrated low levels of TIP at that time, diagnosed in only 1.6% of all peritonitis cases, highlighting either a striking increase in the TIP burden or an earlier underestimation. During the 2014-2015 study, the average age of a TIP patient was 20 years old, and the mortality rate was 6%. Primary repair and resection anastomosis remained the most common surgical treatments. Age over 60 years and anemia were correlated with increased risk of death.

### Tahoua

Presented by Dr. Abdou Souly Aboul Aziz, there were 182 cases of TIP at Tahoua CHR in 2020, comprising 65% of all peritonitis cases and with a mortality rate of 8.2%. A total of 75% of patients were anemic and 66% male. All patients were treated with an ileostomy.

Hôpital de la SIM in Galmi conducted a retrospective review of pediatric TIP patients in 2022, presented by Dr. Yakoubou Sanoussi. There were 191 total patients, with an average age of 7 years old. TIP patients had a 14% mortality rate and typhoid-related surgical disease was attributed to 51% of all pediatric surgical mortality. Additionally, Dr. Katherine Shafer reported trends of TIP at this same hospital in adult women of childbearing age between January 2022 through April 2023. During this time, 28 adult women of childbearing age were operated on for TIP, with a mortality rate of 50%. An average of three children per patient were orphaned because of mortality in this population. Of the three pregnant patients admitted for TIP, spontaneous abortion or early neonatal mortality occurred in all cases.

### Zinder

Dr. Amadou Magagi Ibrahim presented a combined retrospective study from 2014 to 2019 including patients with TIP in Zinder and Maradi. ^9^ During this time, the hospitals had a combined 2,391 TIP cases, comprising 47% of all emergency abdominal surgeries. A total of 72% of the cases occurred in patients under 15 years of age, and 67% of patients hailed from rural regions. Ileostomies were performed in 67% of patients. Mortality rate was 11.2%, with poverty, residence in a rural region, and difficulty accessing healthcare facilities being statistically associated with increased mortality risk. The average cost of hospitalization for TIP patients was approximately 192,500 CFA ($315.83 USD), with costs being much higher for patients with ileostomy creation. ^9^

Using the data detailed above, Dr. Harissou Adamou devised a prognostic score to determine mortality risk of patients presenting with non-traumatic intestinal perforations. Factors that increased the risk of mortality included: presence of a co-morbidity, tachypnea, admission and management delay greater than 72 hours, American Society of Anesthesiology score, serum creatinine level greater than 20 mg/L, hemoglobin less than 9 g/dL, three or more perforations identified in the operating room, and systolic blood pressure under 90 mm/Hg at the time of presentation. This work has since been published in the Journal of the West African College of Surgeons. ^10^

## Typhoid conjugate vaccines

Although typhoid vaccines have long been used by travelers and certain military personnel, as presented by Professor Brah Souleymane, they have yet to become accessible to the local Nigerien people. ^11^ Fortunately, TCVs hold promise for use in typhoid-endemic countries like Niger and have the potential to overcome many of the challenges that impeded uptake of earlier vaccines. In addition to being safe and effective, TCVs provide strong protection for at least four years, ^12^ only require one dose, are safe for children as young as six months, and can be administered in combination with other vaccines. ^13, 14^ Three African countries, Liberia, Zimbabwe, and Malawi, have introduced TCV through campaigns, followed by routine immunization, ^12^ and Burkina Faso is slated to introduce in late 2024. Other countries in Africa and Asia are in the planning phases of TCV applications and introduction.

In the keynote presentation, Dr. Kathleen Neuzil described the continued endemicity of typhoid. Although it has been virtually eliminated from most high-income countries, typhoid remains a significant disease amongst poor and marginalized children with limited access to healthcare. Control of enteric diseases, including typhoid, faces unprecedented challenges due to competing health priorities, increasing political conflicts, economic stress on families and governments, climate change and extreme weather events, and antimicrobial resistance. Recently, fluoroquinolone resistance was identified in Niger through a surveillance study on pediatric TIP patients in Maradi. All isolated typhoid specimens demonstrated fluoroquinolone resistance, concerning in an area where ciprofloxacin remains the first line choice of antibiotic for treatment of febrile children in the outpatient setting. The high numbers of TIP throughout the country and the presence of fluoroquinolone-resistant strains both provide compelling reasons to introduce TCV in Niger.

Dr. Lassane Kabore summarized support for the introduction of TCV. Gavi has pledged to fully fund a one-time single dose catch up campaign for all children up to 15 years of age. Additionally, the organization will co-finance introduction in routine immunization schedules, with each vaccine costing an estimated $0.20 USD (121 CFA with current 2024 exchange rates), with operational grants available for introduction costs. To further emphasize the TCV benefit, a video was shown highlighting the effects of typhoid in children in rural villages in Niger and emphasizing the nominal cost of the vaccine in comparison to the cost of the often multiple surgeries and the subsequent management of disease morbidity, including ostomy care.

## Conclusions

At the first National Typhoid Conference in Niamey, Niger, surgeons from four regions of Niger shared local data that demonstrated TIP remains a major cause of emergency surgery and mortality throughout the country. Across most studies, TIP was responsible for 38 to 65% of acute peritonitis. Additionally, TIP most frequently occurred in patients under 15 years of age and those who reside in rural regions where healthcare is difficult to access. Surgical treatment of TIP depends on severity of disease, particularly number of perforations and location of these perforations. Management can include primary repair, small bowel resection with primary anastomosis, or bowel resection with creation of an ileostomy. Late presentations of TIP often require ileostomy creation which translates to difficulties with management, higher morbidity, and additional costs. Mortality rates associated with TIP remain high, ranging from 11 to 14%, although this is lower than previous case fatality estimates in sub-Saharan African countries. While numbers were small, women of childbearing age had the highest mortality rate amongst all populations presented. In addition to the medical morbidity of TIP, patients and their families also face social hardships secondary to stigma around ostomies and financial hardships due to the high cost of hospitalization and subsequent treatment. In Niger, surgeons provide a unique and important perspective in raising awareness on typhoid and its complications.

As evident by the alarming amount of TIP, the lack of blood culture surveillance in most of Niger should not imply that there is no typhoid fever. While more feasible diagnostics are essential, children should not be denied safe and effective vaccines while such diagnostics are in development and/or blood culture capability is expanded. Thus, countries need to be innovative and flexible in defining disease burden. Additionally, the recent identification of fluoroquinolone-resistant *S.* typhi in Niger is particularly alarming and could lead to catastrophic consequences if not quickly addressed. TCVs offer a single dose, safe, well-tolerated, and cost-effective option. In Niger, TCV could be included as a component of multi-antigen campaigns – for example, with pentavalent meningitis vaccines, yellow fever vaccines, or measles vaccines. Improvements in water, sanitation, and hygiene (WASH) are essential and must not be forgotten. Vaccines and WASH go hand-in-hand to provide short- and long-term tools to prevent and control typhoid. At the first National Typhoid conference in Niger, clinicians and policymakers have come together to take on typhoid and begin to make it a disease of the past.

## Disclaimer

The views expressed in this article are those of the author(s). Publication in VeriXiv does not imply endorsement by the Gates Foundation.

## Data Availability

No data associated with this article.
